# Elevating voices in marine science: an interview with Nikki Traylor-Knowles on coral innate immunity and building scientific community

**DOI:** 10.1038/s42003-022-04149-3

**Published:** 2022-11-09

## Abstract

Dr. Nikki Traylor-Knowles is an Associate Professor of Marine Biology and Ecology at the University of Miami Rosenstiel School of Marine, Atmospheric, and Earth Science. Dr. Traylor-Knowles received her Ph.D. from Boston University and was a NSF Ocean Sciences postdoctoral fellow at Hopkins Marine Station before starting her own research lab in 2016. In this Q&A, Dr. Traylor-Knowles tells us about her work on understanding the complexities of coral and role as the founder of Black Women in Ecology, Evolution, and Marine Science (BWEEMS).

**Figure:**
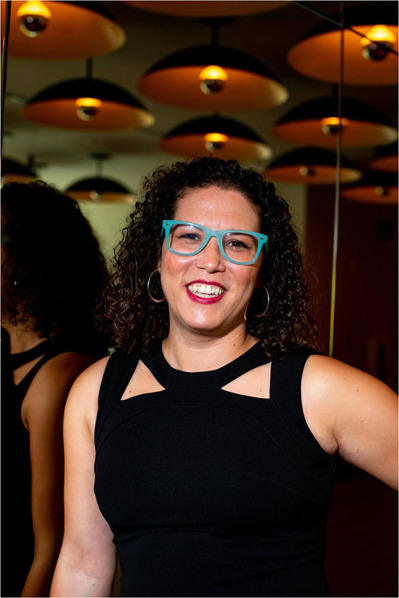
Nikki Traylor-Knowles

Please tell us a little about your academic background and research interests.

I got my B.S. and M.S. degrees from Johns Hopkins University in Cellular and Molecular Biology and my Ph.D. from Boston University in Biology. I was a NSF Ocean Science Postdoctoral fellow at Hopkins Marine Station at Stanford University. In 2016, I started as an Assistant Professor at University of Miami Rosenstiel School of Marine, Atmospheric, and Earth Science and was recently awarded tenure and promotion (yay!). In general, I am interested in the evolution, mechanisms, and function of the immune system in early diverging metazoans including the phyla Cnidaria (sea anemones, stony corals) and Ctenophora (comb jellies).

Cnidarians are increasingly at risk due to the effects of climate change. How does your group’s research tie into conservation or restoration efforts for coral?

One of the areas my laboratory is exploring is how coral’s innate immune system is affected by climate change. Climate change is a broad term that encompasses many stressors, but my lab is primarily focused on thermal stress, as well as disease (which is linked to stress in the environment). We use clinical methods, such as fluorescence-activated cell sorting and genomics to understand how the cellular immune system of corals is reacting to changes in their environment. We use this information to develop monitoring systems for the coral innate immune system. Additionally, we use genomics to understand how different coral colonies are reacting to disease and stress, which can be integrated into restoration plans.

Did the pandemic impact your ability to conduct field work or collect samples?

The biggest effect was that we had to really reduce the amount of people in the lab and offices, so there was a lot of coordination initially to make sure that we could do this. Field collection was very slow in the beginning of the pandemic due to restrictions on boats, etc., but we just pivoted and focused on analyzing the data that we did have, and process samples that were in our freezer.

Changing gears; you also founded Black Women in Ecology, Evolution, and Marine Science (BWEEMS). Could you tell us a little about the mission for BWEEMS and its ongoing programming?

Our mission at BWEEMS is to make a global community that encourages the autonomy of Black women to push boundaries, drive innovation and elevate our voices in evolution, ecology and marine science. Our vision is to establish a Black women-led community to elevate and support other Black women in Ecology, Evolution and Marine Science (EEMS). We are cultivating our own narrative. We can be our authentic selves, embracing our diverse cultures, backgrounds, and perspectives, while leading, thriving, and innovating. Our community values include: (1) Elevating Black women’s voices and innovations, (2) Inspiring sisterhood through community and collaboration, (3) Driving equitable spaces, leadership, and science, (4) Catalyzing a paradigm shift in EEMS spaces, and (5) Promoting a culture of acceptance by providing black women non-judgmental opportunities to come as they are and stand in their truth. We have monthly programming that is free to all BWEEMS members (membership is free as well) which includes professional development, networking, mentorship, community meet-ups and wellness. Our membership ranges from undergraduates to retired professionals. We embrace the diversity of Black women at all career stages within EEMS.

What advice would you give to scientists trying to build similar communities to BWEEMS?

Go slow with it and be guided by what your community needs, not just by what you need. Don’t immediately go to starting a non-profit, but instead start with community meetings where you can listen and gather the data needed to then move forward.

How do you maintain an inclusive environment in your own research lab?

In my lab we embrace a culture of openness and respect. I make sure that space is made for everyone to be heard and that differing opinions are not a bad thing. I am intentional about who I choose to work with in science, and I make it clear that we do not accept any type of “-ism” in my lab. Lastly, I approach mentorship as an open space where my lab members can come to me and be honest. I am clear that I am not a therapist, but rather that I am there to help and connect. I can’t read their minds, so I need open communication. I think that this helps to create a space for students to be themselves while still being professional.

Last but not least; what is something you wish more people knew about coral?

I wish that more people knew that corals are COMPLEX. They have a complex innate immune system and they have complex symbioses that are crucial for their survival. Lastly, I wish that people understood how important coral reefs are for coastal protection. We love our beaches, and if we want to keep them, we need to have coral reefs.

*This interview was conducted by Senior Editor George Inglis*.

